# Cytonuclear Epistasis Controls the Density of Symbiont *Wolbachia pipientis* in Nongonadal Tissues of Mosquito *Culex quinquefasciatus*

**DOI:** 10.1534/g3.117.043422

**Published:** 2017-06-09

**Authors:** Kevin J. Emerson, Robert L. Glaser

**Affiliations:** * Department of Biology, St. Mary’s College of Maryland, Maryland 20686-3001; † Wadsworth Center, New York State Department of Health, Albany, New York 12201-0509; ‡ Department of Biomedical Sciences, University at Albany, New York 12222

**Keywords:** cytonuclear epistasis, *Wolbachia pipientis*, *Culex quinquefasciatus*, *Wolbachia* density, QTL mapping

## Abstract

*Wolbachia pipientis*, a bacterial symbiont infecting arthropods and nematodes, is vertically transmitted through the female germline and manipulates its host’s reproduction to favor infected females. *Wolbachia* also infects somatic tissues where it can cause nonreproductive phenotypes in its host, including resistance to viral pathogens. *Wolbachia*-mediated phenotypes are strongly associated with the density of *Wolbachia* in host tissues. Little is known, however, about how *Wolbachia* density is regulated in native or heterologous hosts. Here, we measure the broad-sense heritability of *Wolbachia* density among families in field populations of the mosquito *Culex pipiens*, and show that densities in ovary and nongonadal tissues of females in the same family are not correlated, suggesting that *Wolbachia* density is determined by distinct mechanisms in the two tissues. Using introgression analysis between two different strains of the closely related species *C. quinquefasciatus*, we show that *Wolbachia* densities in ovary tissues are determined primarily by cytoplasmic genotype, while densities in nongonadal tissues are determined by both cytoplasmic and nuclear genotypes and their epistatic interactions. Quantitative-trait-locus mapping identified two major-effect quantitative-trait loci in the *C. quinquefasciatus* genome explaining a combined 23% of variance in *Wolbachia* density, specifically in nongonadal tissues. A better understanding of how *Wolbachia* density is regulated will provide insights into how *Wolbachia* density can vary spatiotemporally in insect populations, leading to changes in *Wolbachia*-mediated phenotypes such as viral pathogen resistance.

*Wolbachia pipientis* Hertig is an intracellular, gram-negative, α-proteobacterial symbiont that infects arthropods and nematodes, including 40–60% of insect species (Hilgenboecker *et al.* 2008; Schneider *et al.* 2012; Zug and Hammerstein 2012; Sicard *et al.* 2014). It is a reproductive parasite that is vertically transmitted through the female germline and can manipulate aspects of its host’s reproduction to favor the preferential survival of infected females through means such as male killing, parthenogenesis, male feminization, and, most frequently, cytoplasmic incompatibility (Werren *et al.* 2008). With cytoplasmic incompatibility, mating between infected males and uninfected females results in reduced embryonic viability of the progeny, while the reciprocal mating between uninfected males and infected females results in normal embryonic development of progeny that are now *Wolbachia* infected. In addition to infecting the germline, *Wolbachia* can also infect somatic tissues, where it can cause nonreproductive phenotypes in its host, including nutrient provisioning and pathogen resistance (Dobson *et al.* 1999; Hedges *et al.* 2008; Teixeira *et al.* 2008; Brownlie *et al.* 2009; Hosokawa *et al.* 2010; Sicard *et al.* 2014; Moriyama *et al.* 2015).

In recent years, substantial research has focused on exploiting the unique biological characteristics of *Wolbachia* to develop *Wolbachia* infection of vector mosquitoes as an approach for interrupting the transmission cycle of disease pathogens (Bourtzis *et al.* 2014; Caragata *et al.* 2015; Hoffman *et al.* 2015). Establishing stable *Wolbachia* infections in heterologous, nonnative hosts via transinfection leads to *Wolbachia*-mediated phenotypes in the new host. These phenotypes can both drive *Wolbachia* infection through a naive, uninfected population via the cytoplasmic-incompatibility phenotype, while at the same time, reducing the host’s susceptibility to infection by disease pathogens via the pathogen-resistance phenotype. For example, this ability has been exploited to create *Aedes aegypti* mosquitoes infected with the *w*Mel strain of *Wolbachia* from *Drosophila melanogaster*. Release of these mosquitoes into the wild can spread the *Wolbachia* infection into uninfected *A. aegypti* populations, making the mosquitoes less able to be infected by, and transmit, significant viral pathogens such as Dengue, Chikungunya, and Zika viruses, with the goal of ultimately reducing the incidence of disease (Moreira *et al.* 2009; Frentiu *et al.* 2014; Dutra *et al.* 2016).

Many *Wolbachia*-mediated host phenotypes are associated with *Wolbachia* density (Breeuwer and Werren 1993; Jaenike 2009; Unckless *et al.* 2009). This has been particularly well documented for pathogen-resistance phenotypes, where higher densities in somatic tissues are correlated with stronger pathogen resistance (Osborne *et al.* 2009, 2012; Martinez *et al.* 2014). Higher *Wolbachia* densities, however, can also be associated with reduced host fitness, including reduced embryonic, larval, and host viability, as well as reduced fertility and fecundity (Min and Benzer 1997; Duron *et al.* 2006; Martinez *et al.* 2015). Successful utilization of *Wolbachia* infection as a biocontrol agent requires a trade-off between infection densities that are high enough to provide robust pathogen resistance, but not so high as to reduce host fitness to a point where *Wolbachia* infection is lost from a population. For example, even in the presence of a reproductive-drive phenotype like cytoplasmic incompatibility, *A*. *aegypti* infected with high densities of the *w*MelPop strain of *Wolbachia* from *D*. *melanogaster* show low fitness both in the laboratory and when released into natural populations, limiting the efficacy of release projects (Min and Benzer 1997; Chrostek *et al.* 2013; Nguyen *et al.* 2015).

The control of *Wolbachia* density is complex, involving, to varying extents in different systems, the *Wolbachia* genotype (Min and Benzer 1997; Mouton *et al.* 2003; Dutton and Sinkins 2004; Tortosa *et al.* 2010; Martinez *et al.* 2017); host genotype (Ikeda *et al.* 2003; Kondo *et al.* 2005; Duron *et al.* 2006; Mouton *et al.* 2007); environmental effects, like temperature (Stouthamer *et al.* 1990; Clancy and Hoffman 1998; Johanowicz and Hoy 1998; Van Opijnen and Breeuwer 1999; Hurst *et al.* 2000; Mouton *et al.* 2006); aspects of host physiology, including age (Berticat *et al.* 2002; Unckless *et al.* 2009) and nutritional status (Clancy and Hoffman 1998; Dutton and Sinkins 2004; Serbus *et al.* 2015); and interactions among these various factors (Dutton and Sinkins 2004; Kondo *et al.* 2005; Mouton *et al.* 2007; Carrington *et al.* 2009). Given this complexity, it is not surprising that *Wolbachia* densities can vary significantly in the field between individual insects from a given population (Berticat *et al.* 2002; Hoffman *et al.* 2014), with density differences between individuals sometimes exceeding 20,000-fold (Unckless *et al.* 2009; Sumi *et al.* 2017).

The underlying molecular mechanisms that ultimately determine *Wolbachia* density in any given *Wolbachia*–host system are poorly understood in native hosts (Voronin *et al.* 2012; Newton *et al.* 2015; White *et al.* 2017b), and even less well understood in transinfected heterologous hosts, where *Wolbachia* replication control is often lost (Boyle *et al.* 1993; McGraw *et al.* 2002). Even in the well-studied system of the *w*MelPop strain of *Wolbachia* in *D. melanogaster*, it has proven difficult to identify the genetic variations in the *Wolbachia* genome that cause the overreplication, high-density phenotype displayed by *w*MelPop (Chrostek *et al.* 2013; Chrostek and Teixeira 2015; Rohrscheib *et al.* 2016). A better understanding of the regulation of *Wolbachia* density, and the roles played by the *Wolbachia* and host genomes in that regulation, would provide much needed insight into how *Wolbachia* density in insect populations can change across space and time, leading to changes in *Wolbachia*-mediated phenotypes like pathogen resistance.

*Culex pipiens* L. and *C. quinquefasciatus* Say are sibling species within the *C. pipiens* species complex of mosquitoes. They are primary vectors for West Nile virus in the northeastern and southeastern United States, respectively, with a large zone of fertile hybrids in between (Turell *et al.* 2000; Bernard *et al.* 2001; Fonseca *et al.* 2004; Kilpatrick *et al.* 2005; Harbach 2012). Both species are naturally infected by the *w*Pip strain of *Wolbachia* (Hertig and Wolbach 1924; Cornel *et al.* 2003; Rasgon and Scott 2003; Micieli and Glaser 2014). The natural *w*Pip infection of *C*. *quinquefasciatus* can confer a pathogen-resistance phenotype on its host, and has been shown to reduce the competence of *C*. *quinquefasciatus* to be infected by, and transmit, West Nile virus (Glaser and Meola 2010).

Previously, we showed that *Wolbachia* densities in whole and ovariectomized, field-collected *C. pipiens* vary between mosquito families (Micieli and Glaser 2014). Since a large majority (>99%) of *Wolbachia* measured in whole *C*. *pipiens* female mosquitoes is located in the ovary, this observation suggested that variation in *Wolbachia* densities in ovary (whole mosquitoes) and nongonadal tissues (ovariectomized mosquitoes) are determined, at least in part, by genetic variation between families. In this report, we extend those observations, showing that the density of *Wolbachia* in whole *vs.* ovariectomized mosquitoes in each *C*. *pipiens* family are not correlated and that broad-sense heritability explains a significant fraction of the variation in *Wolbachia* density across families. These observations suggest that control of *Wolbachia* densities in the ovary and nongonadal tissues of *C*. *pipiens* is determined by independent genetic contributions in each tissue. In support of this hypothesis, we show by introgression analysis that *Wolbachia* densities in whole *C*. *quinquefasciatus* mosquitoes are determined primarily by cytoplasmic genotype, while *Wolbachia* densities in ovariectomized mosquitoes are determined by both nuclear and cytoplasmic genotype and their epistatic interactions. Finally, we identify quantitative-trait loci (QTL) in the host *C*. *quinquefasciatus* genome that affect *Wolbachia* density specifically in nongonadal tissues.

## Materials and Methods

### Heritability analysis

Collection of the data on *Wolbachia* density in families of *C. pipiens* has been described previously (Micieli and Glaser 2014). Briefly, *C*. *pipiens* egg rafts were collected from the field from numerous sites in and around Albany, NY. The larvae from individual rafts were reared to adulthood under standardized conditions, minimizing impact of environmental variables on *Wolbachia* density. We extracted DNA from individual whole or ovariectomized females to quantify *Wolbachia* densities in the ovary and nongonadal tissues, respectively. Whole females can be used as a proxy for measuring *Wolbachia* in the ovary because a large majority (>99%) of *Wolbachia* measured in whole *C*. *pipiens* female mosquitoes is located in the ovary (Micieli and Glaser 2014). We measured *Wolbachia* density by quantitative real-time PCR, measuring the number of *Wolbachia wsp* gene sequences relative to the number of mosquito *ribosomal protein L32* (*RpL32*) gene sequences. The copy number of *wsp* gene sequences was divided by the copy number of *RpL32* gene sequences in each sample to calculate relative *Wolbachia* density (Micieli and Glaser 2014). Broad-sense heritability was calculated as the proportion of the total variance attributable to the among-family component of variance (Roff 1997). We used Pearson’s correlation test to compare *Wolbachia* densities in the ovary and somatic tissues across all the families.

### Introgression analysis

The Ben95 and Arg12 strains of *C*. *quinquefasciatus*, their differing levels of *Wolbachia* in ovary and nongonadal tissues, and the conditions used for their rearing and maintenance have been described previously (Micieli and Glaser 2014). We set up reciprocal crosses between the two mosquito strains, each cross with ∼200 virgin females from one strain crossed to ∼200 males from the other strain. After 1 wk of mating, females were fed on chicken blood, egg rafts collected after 1 wk, and the larvae reared to adulthood. Approximately 200 virgin female F1 progeny were then collected from each of the two crosses and backcrossed to ∼200 males from the same strain from which males were used in the initial parental cross. We repeated this backcross four times, until the F5 generation, at which point introgression reaches 97%. *Wolbachia* density in the ovary or in nongonadal tissues was measured in individual females collected from the parental colonies and from the F1 and F5 generations as described previously (Micieli and Glaser 2014). We used two-way ANOVA to analyze differences in *Wolbachia* densities in the parental and F5 generations among cytoplasmic and nuclear genotypes and to test for cytonuclear epistasis.

### Genetic mapping panel

The mapping cross consisted of a single Arg12 female and a single Ben95 male *C*. *quinquefasciatus* (Supplemental Material, File S1). The F1 larvae produced by the parental female were reared to adulthood, and the F1 full-sibling adults allowed to mate *inter se* before being fed on chicken blood. F2 egg rafts were collected, and the larvae reared to adulthood. Females used for genetic mapping were randomly chosen from the F2 population when they were 3–5 d old. Each F2 female was ovariectomized, and the carcass collected and stored for later genotyping and phenotype analysis. DNA was isolated from the parental male, parental female, and 91 ovariectomized F2 hybrid females. The DNA was used for both commercial nextRAD sequencing (SNPsaurus, LLC, Eugene, OR) (Russello *et al.* 2015) and measuring relative *Wolbachia* density in nongonadal tissues as described previously (Micieli and Glaser 2014).

### SNP genotyping

All genotyping was performed using the RADseq analysis pipeline STACKS v. 1.4 (Catchen *et al.* 2011, 2013). Raw 101-bp Illumina reads were quality filtered with the STACKS component *process_radtags* using default parameters. Quality-filtered reads were then aligned to the *C*. *quinquefasciatus* genome assembly CpipJ2 (Arensburger *et al.* 2010) using the aligner GSNAP (Wu and Watanabe 2005; Wu and Nacu 2010) with the parameter min-coverage set to 0.9. Individual alignment files were then processed using the *ref_map.pl* wrapper script for STACKS (database details available in File S2). Genotype corrections were performed using the STACKS component *genotypes* (final genotype calls available in File S3).

### Linkage mapping

A total of 2735 nextRAD loci that were divergent across parents (aa/bb or ab/cd type markers) were identified, of which 952 were genotyped in at least 80 of the 91 offspring and were included in the downstream analysis. Linkage mapping was performed primarily using R/qtl (Broman *et al.* 2003; Broman and Sen 2009; Broman 2015) with map distances calculated using the Kosambi map function (Kosambi 1943) and a genotype error probability of 0.01. Marker order was first determined using a modified *orderMarkers* function that invoked a *ripple* function after the addition of every 10 markers (File S1). Some manual curation of the marker order was performed to optimize the likelihood of the resulting linkage groups (LGs) (File S1). After creation of the linkage map, genotypes that were the result of a double crossover event surrounding a single marker and loci with strong segregation distortion were removed. The 779 retained loci were positioned at 246 map locations in the final map (these are the “bin marker” positions shown in Figure 3, Figure S2, and Figure S3). Chromosome lengths were calculated using the methods of Chakravarti *et al.* (1991) and Fishman *et al.* (2001). Marker dispersion was assessed using a one-dimensional, nearest-neighbor test for each LG (Clark and Evans 1954).

### QTL mapping

Standard interval mapping implemented in R/qtl was used to identify QTL in *C*. *quinquefasciatus* that influence *Wolbachia* density in nongonadal tissues (Broman *et al.* 2003; Broman and Sen 2009). QTL LOD scores were estimated using extended Haley–Knott regression in the R/qtl function *scanone*. Significance levels were estimated via 1002 permutations using the same function.

### Data availability

Strains are available upon request. File S1 contains additional details of the material and methods. File S2 contains summary data from (1) the nextRAD, STACKS, and phenotype analysis of each F2 hybrid; (2) the linkage mapping analysis; and (3) the genomic scaffolds identified by the genetic linkage analysis. File S3 contains the vcf file of genotype calls for each nextRAD locus from the STACKS analysis. File S4 contains *R* scripts used for the linkage mapping analysis. The raw 101-bp Illumina reads have been placed in the NCBI Short Read Archive (BioProject ID PRJNA378432).

## Results

### Heritability of Wolbachia density in C. pipiens

Herein, we use *Wolbachia* density in whole females as a proxy for measuring *Wolbachia* density in the ovary, because a large majority (>99%) of *Wolbachia* measured in whole *C*. *pipiens* female mosquitoes is located in the ovary, while ovariectomized mosquitoes provide a measure of *Wolbachia* density solely in nongonadal tissues (Micieli and Glaser 2014).

Previously, we demonstrated that in field populations of *C*. *pipiens* mosquitoes, familial variation in *Wolbachia* density in both whole and ovariectomized female mosquitoes behaves as a complex quantitative trait (Micieli and Glaser 2014). This variation among individuals in *Wolbachia* density is likely due, at least in part, to genetic variation between the different mosquito families because the measurements were made on mosquitoes that had been reared from field-collected egg rafts under standardized conditions of temperature, density, and nutrition, thereby removing major sources of environmental variation that might impact *Wolbachia* density.

**Figure 1:**
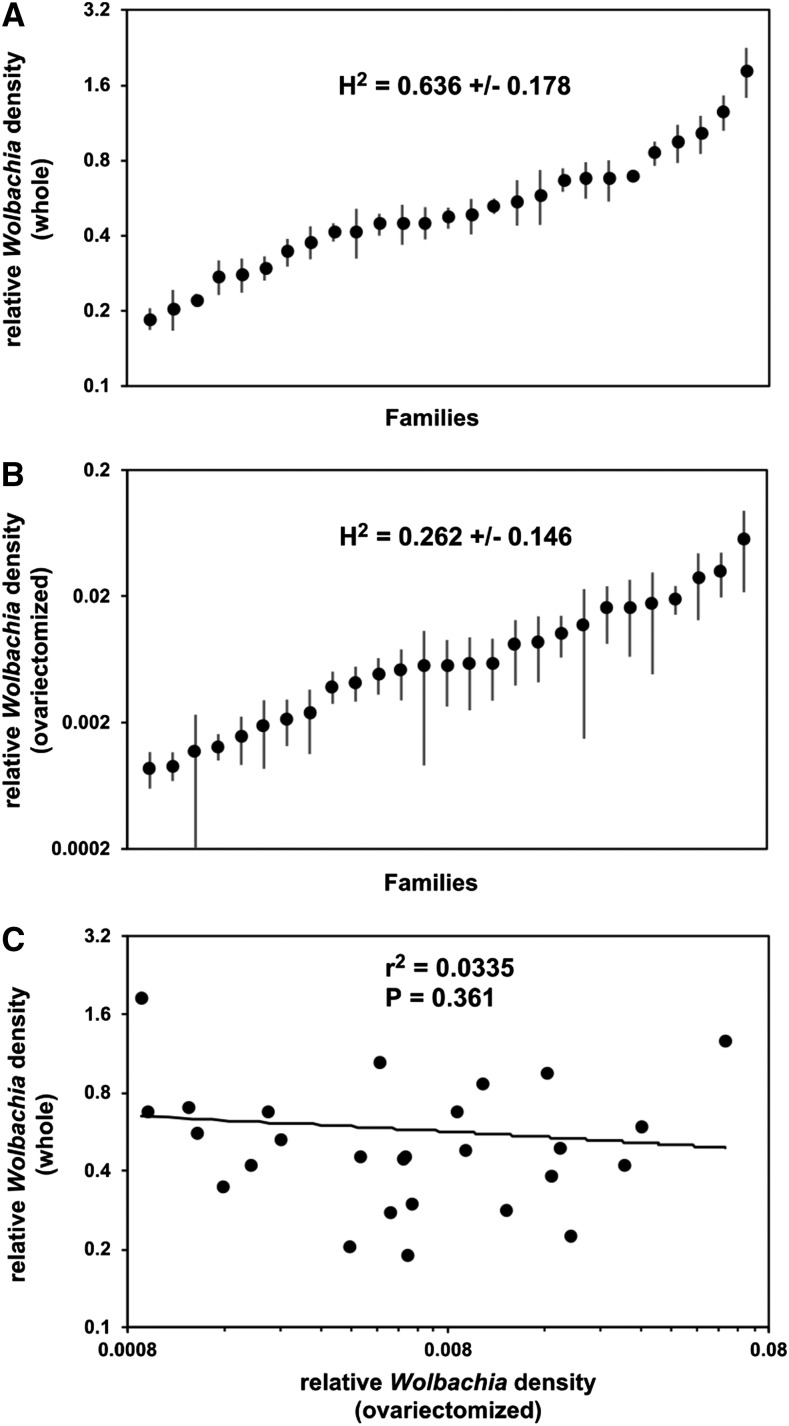
Heritability analysis of *C*. *pipiens*. Rank ordering of field-collected *C*. *pipiens* families by mean *Wolbachia* density in (A) whole females, with most (>99%) of the measured *Wolbachia* from the ovary, and (B) ovariectomized females, with the measured *Wolbachia* from nongonadal tissues. The mean (±SE) for six siblings from each family is shown. Levels of broad-sense heritability are shown with SE estimates. (C) Pearson’s correlation test of *Wolbachia* densities in ovary *vs.* nongonadal tissues. Data used for calculations were originally reported in Micieli and Glaser (2014).

We tested the idea that genetics plays a role in driving variation in *Wolbachia* density in *C*. *pipiens* mosquitoes by reexamining the data from Micieli and Glaser (2014) and measuring the broad-sense heritability of the *Wolbachia* density phenotype. *Wolbachia* density in whole mosquitoes (ovary tissues) had high broad-sense heritability (*H*^2^ = 0.636 ± 0.178 SE), while *Wolbachia* density in ovariectomized mosquitoes (nongonadal tissues) had a relatively high, though not significantly different than zero, level of heritability (*H*^2^ = 0.262 ± 0.146 SE) (Figure 1, A and B). This demonstrates that genetic variation does contribute to variation in *Wolbachia* density in both ovary and nongonadal tissues of *C*. *pipiens* mosquitoes. Lastly, we also compared the densities of *Wolbachia* in whole *vs.* ovariectomized mosquitoes for each *C*. *pipiens* family and discovered that the densities are not correlated (*r*^2^ = 0.03, *P* = 0.38; Figure 1C). This result suggests that the genetics underlying variation of *Wolbachia* density are different in ovary *vs.* nongonadal tissues.

### Genetic control of Wolbachia density in C. quinquefasciatus

**Figure 2:**
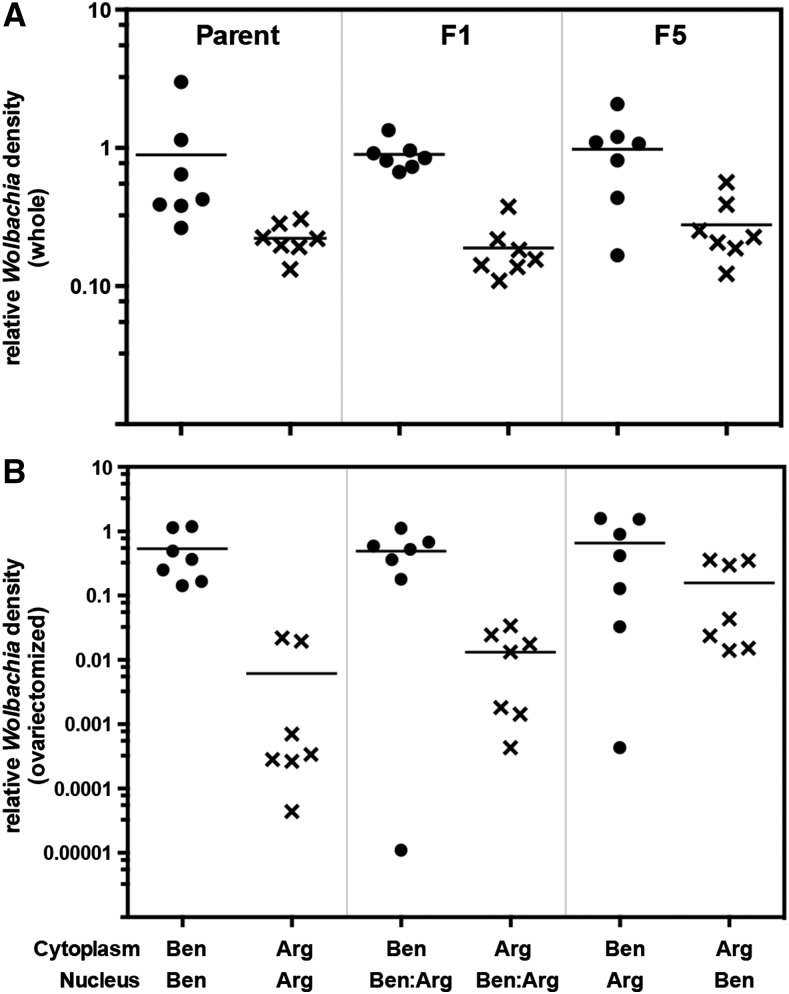
Introgression analysis of *C*. *quinquefasciatus*. Reciprocal introgression backcrosses were performed between the Ben95 (Ben) and Arg12 (Arg) strains of *C*. *quinquefasciatus*. *Wolbachia* densities were measured in (A) whole females, with most (>99%) of the measured *Wolbachia* from the ovary, and (B) ovariectomized females, with the measured *Wolbachia* from nongonadal tissues. Female mosquitoes containing either Ben95-derived (•) or Arg12-derived (×) cytoplasmic genotypes are indicated. Measurements were made on mosquitoes with the parental, F1, and F5 nuclear genotypic background, as indicated at the top of (A). The cytoplasmic and nuclear genotypes for the mosquitoes collected for each sample are indicated across the bottom of (B). Means are indicated by a horizontal line.

Familial variation in any phenotype can be caused by genetic variation potentially originating from two different sources, the nuclear genotype as well as the maternally inherited cytoplasmic genotype, which in this case consists of the *Wolbachia* and mitochondrial genomes. We considered the possibility that *Wolbachia* densities in whole *vs.* ovariectomized mosquitoes vary independently because of the differing influence of nuclear *vs.* cytoplasmic genotype on *Wolbachia* density in ovary *vs.* nongonadal tissues. Testing this possibility using field-collected *C*. *pipiens*, however, was not feasible. Instead, we used two colonized strains of *C*. *quinquefasciatus* mosquitoes to directly test the influence of nuclear *vs.* cytoplasmic genotype on *Wolbachia* density in whole *vs.* ovariectomized mosquitoes (Figure 2). The Ben95 and Arg12 strains of *C*. *quinquefasciatus* used for the analysis have consistent differences in *Wolbachia* density, with the Ben95 mosquitoes having significantly higher densities in both whole and ovariectomized mosquitoes (Micieli and Glaser 2014) (Figure 2).

We used reciprocal-introgression backcrosses between the Ben95 and Arg12 strains of *C*. *quinquefasciatus* to introgress the maternally transmitted cytoplasmic genotype of each strain into the nuclear genotypic background of the alternate strain, and then measured *Wolbachia* densities in whole (ovary tissues) *vs.* ovariectomized (nongonadal tissues) mosquitoes (Figure 2). There was not a significant effect of nuclear genotype on *Wolbachia* density in whole mosquitoes (*F*_1,24_ = 3.39, *P* = 0.078) (Table S1), although the limited statistical power of the analysis combined with a relatively low *P* value means that a weak effect of nuclear genotype cannot be excluded. In contrast, there was a very strong effect of cytoplasmic genotype (*F*_1,24_ = 22.57, *P* = 8 × 10^−5^), with Ben95-derived cytoplasm associated with higher *Wolbachia* densities than Arg12-derived cytoplasm.

We found evidence for cytonuclear epistasis (cytoplasmic- by nuclear-genotype interaction) in the determination of *Wolbachia* densities in ovariectomized mosquitoes (*F*_1,24_ = 4.83, *P* = 0.038), along with both cytoplasmic (*F*_1,24_ = 19.42, *P* = 1.8 × 10^−4^) and nuclear (*F*_1,24_ = 11.83, *P* = 0.002) genotypic effects (Figure 2B). *Wolbachia* densities among ovariectomized mosquitoes having both Arg12 nuclear and cytoplasmic genotypes were significantly lower than all other genotypic combinations (Tukey HSD test, *P* < 0.01 in all cases).

### QTL controlling Wolbachia density in nongonadal tissues

The results of the introgression analysis suggest that the nuclear genomes of the Arg12 and Ben95 *C*. *quinquefasciatus* mosquitoes contain QTL that control, through cytonuclear interactions, the density of Arg12-derived *Wolbachia* in nongonadal tissues. This results in low densities in ovariectomized mosquitoes with an Arg12 nuclear genotype, and high densities in ovariectomized mosquitoes with a Ben95 nuclear genotype (Figure 2B). Given that *Wolbachia* density in nongonadal tissues is a primary factor determining the strength of pathogen-resistance phenotypes provided by *Wolbachia* infection, identifying the genes underlying the predicted QTL would provide insight into the nature of *Wolbachia*–host interactions in the *w*Pip–*Culex* system that influence *Wolbachia* density. As an initial step in that effort, we identified QTL in the *C*. *quinquefasciatus* genome that control the difference in *Wolbachia* densities in nongonadal tissues between the Arg12 and Ben95 mosquitoes.

**Figure 3:**
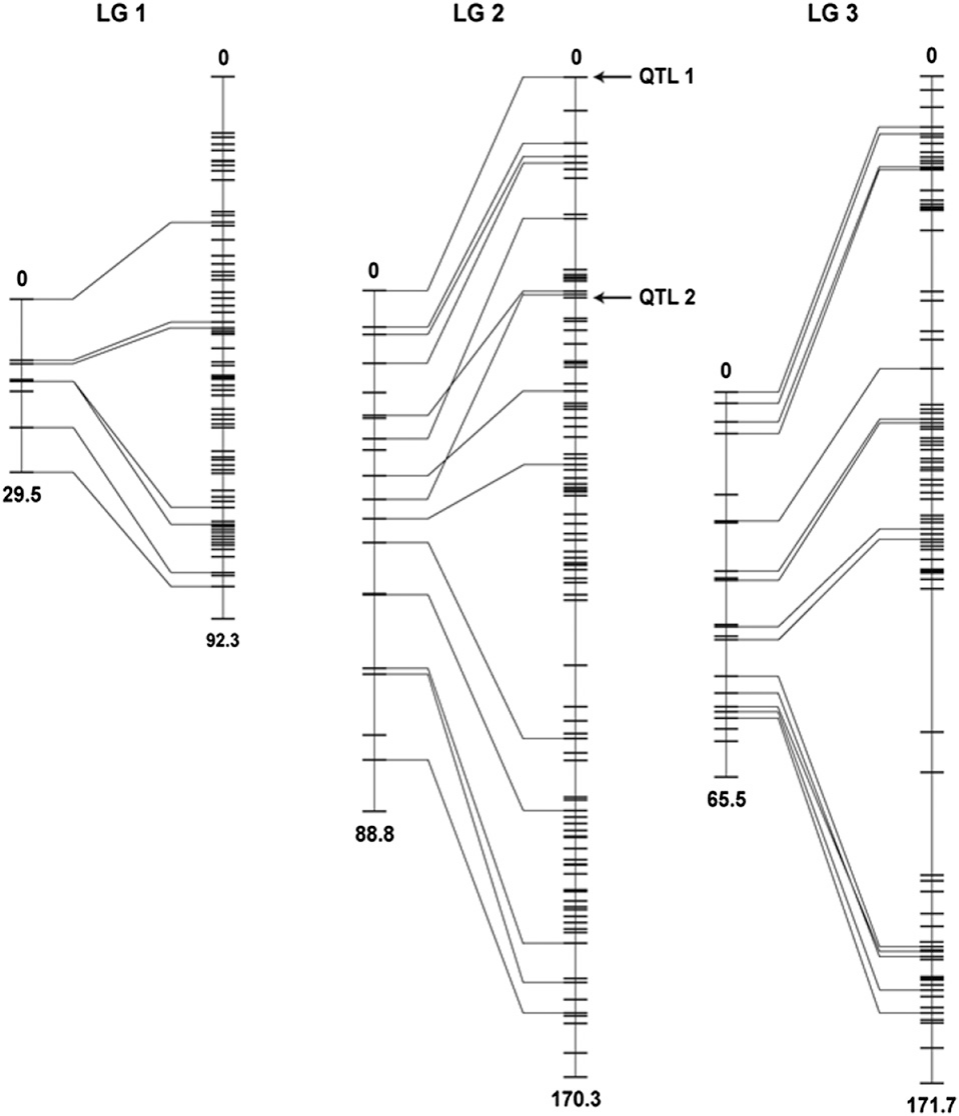
*C. quinquefasciatus* genetic linkage maps. The SNP-based linkage maps reported here (right) are compared to microsatellite-based linkage maps reported previously (left)(Hickner *et al.* 2013). Lines between the maps connect markers located on the same genomic scaffolds shared between the two maps. Total chromosome lengths in centimorgan and the positions of QTL 1 and QTL 2 are indicated.

We performed the QTL analysis using an F2 mapping population that was created by crossing a single Arg12 *C*. *quinquefasciatus* female to a single Ben95 male. *Wolbachia* densities in nongonadal tissue of the F2 mosquitoes were confirmed to span the full range of densities observed in the parental strains, providing the phenotypic variance needed for QTL mapping (Figure S1). DNA was extracted from the parent mosquitoes and 91 ovariectomized F2 females and used for SNP genotyping and measurement of *Wolbachia* density. SNP genotyping was done using nextRAD sequencing (Russello *et al.* 2015), and the genotypes at 779 genomic locations were used to construct linkage maps of the three chromosomes in the *C*. *quinquefasciatus* genome (Figure 3, File S1, File S2, File S3, File S4, and Table S2). This represents the highest-resolution genetic linkage map for *C. quinquefasciatus* to date, with ∼10-fold more markers than previous maps (Hickner *et al.* 2013). The linear order of SNP-based markers in each LG was in good agreement with the order of microsatellite-based markers in the earlier maps, with only a couple of marker locations in disagreement (Figure 3). Localized differences in recombination density between the current and earlier maps are not unexpected given that comparable strain-specific, localized differences in recombination density have been reported in other insect species (Comeron *et al.* 2012), and the LG lengths reported here are also very similar to the LG sizes reported for the related mosquito *C*. *pipiens pallens* (Zou *et al.* 2015).

**Figure 4:**
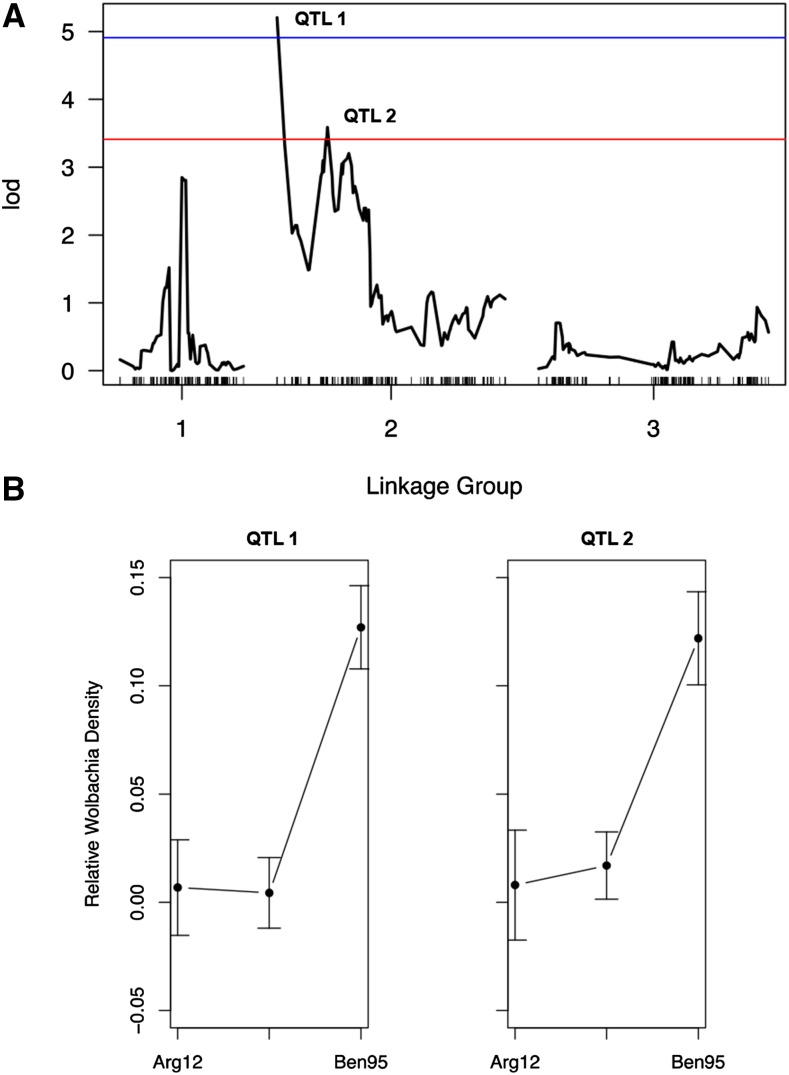
A QTL scan for *Wolbachia* density in nongonadal tissues. The genome was scanned for QTL using the SNP genotypes and *Wolbachia*-density measurements made for 91 F2 females from the mapping population. (A) Two major-effect QTL were identified on LG 2. The 0.1 (red) and 0.05 (blue) whole-genome significance thresholds are shown. (B) Genetic effect analysis with means and SEs shown for *Wolbachia* density in nongonadal tissue measured in mosquitoes homozygous and heterozygous for QTL alleles from the indicated parental strain.

We identified two major-effect QTL on LG 2 at positions 0 cM (LOD 5.20), and 37.6 cM (LOD 3.59), explaining a combined 23% of the variance in nongonadal *Wolbachia* density (Figure 4A; Haley–Knot regression test, *P* = 7.3 × 10^−5^). Although QTL located at the ends of chromosomes can be problematic due to reduced mapping accuracy, the position of QTL 1 is based on three nextRAD markers located on two genomic scaffolds (File S2 and Table S3). All three markers map to position 0 cM with higher confidence than any other position on LG 2 (ΔLOD compared to next best position = 24), suggesting that the location of the markers, and of QTL 1, is accurate. The position of QTL 2 is based on seven markers located on four genomic scaffolds (File S2 and Table S3). The QTL reflect loci with recessive alleles in the Ben95 *C*. *quinquefasciatus* genome, with *Wolbachia* densities being significantly higher in mosquitoes homozygous for the Ben95 allele at each of the QTL than in mosquitoes homozygous for the Arg12 alleles or in heterozygotes (Figure 4B; ANOVA, Tukey HSD, *P* < 0.001 for both QTL).

## Discussion

### Cytonuclear epistasis

The introgression analysis suggests that *Wolbachia* densities in the ovary *vs.* nongonadal tissues of *C*. *quinquefasciatus* are determined, in part, by distinct genetic mechanisms. The cytoplasmic genome plays the predominant role in determining *Wolbachia* density in the ovary, while both the cytoplasmic and nuclear genomes, and their interactions, determine *Wolbachia* density in nongonadal tissues (Figure 2). *Wolbachia* is most likely the major phenotypic driver underlying the cytoplasmic genotype, although a role for mitochondria cannot be excluded. Furthermore, the role of the host nuclear genome in determining *Wolbachia* density in nongonadal tissues varies depending on cytoplasmic genotype, with the Arg12-derived cytoplasmic genotype being more sensitive to nuclear genotypic background than Ben95 (Figure 2B).

These observations in *C*. *quinquefasciatus* support our original supposition that *Wolbachia* densities in whole *vs.* ovariectomized field-collected *C*. *pipiens* vary independently because of the differing influence of nuclear *vs.* cytoplasmic genotype on *Wolbachia* density in ovary *vs.* nongonadal tissues (Figure 1C). The lack of correlation would be consistent with *Wolbachia* density in the ovaries of *C*. *pipiens* varying between families primarily due to variation in cytoplasmic genotype, and *Wolbachia* density in nongonadal tissues varying between families primarily due to variation in, and epistasis between, both cytoplasmic and nuclear genotype. Because the *C*. *pipiens* families were field-collected as egg rafts and reared under standardized conditions, environmental effects were unlikely to have had much impact on the *Wolbachia* densities measured in laboratory-reared mosquitoes. A lack of environmental effects may have also facilitated detection of the genetic effects identified here. This does not exclude the possibility, however, that environmental factors and maternal effects impact *Wolbachia* densities in *C*. *pipiens* that develop entirely in the field, especially given the effects of environmental and physiological factors that have been demonstrated in other *Wolbachia*–host systems (see *Introduction*). Finally, field populations of *C*. *pipiens* have low densities of *Wolbachia* in nongonadal tissues, comparable to the low densities observed in Arg12 *C*. *quinquefasciatus* and unlike the high densities in Ben95 *C*. *quinquefasciatus* (Micieli and Glaser 2014). Based on this similarity, *Wolbachia* densities in nongonadal tissues of *C*. *pipiens* are likely to be sensitive to cytonuclear genetic epistasis like that observed for the Arg12-derived cytoplasmic genotype in *C*. *quinquefasciatus*.

The separation of genetic influence on *Wolbachia* density between the cytoplasmic and nuclear genomes has implications for understanding the sources of, and evolutionary pressures on, *Wolbachia*-mediated phenotypes in these *Culex* species of mosquito. Based on the results reported here, gonad-related phenotypes, like maternal transmission and reproductive-drive mechanisms, may more often be influenced by the cytoplasmic genotype, including *Wolbachia* genetics; while phenotypes originating in nongonadal tissues, like pathogen resistance, may more often be influenced by mosquito genetics. So, within any given *C*. *pipiens* or *C*. *quinquefasciatus* population, genetic variation in both the *Wolbachia* and host genomes, and the interactions between those genomes, need to be considered to fully understand how genetic variation can drive density-dependent *Wolbachia* phenotypes.

Finally, the extent to which a similar separation between cytoplasmic and nuclear genotypes influences tissue-specific *Wolbachia* density in other *Wolbachia*–hosts systems is unknown. In most studies, *Wolbachia* density is measured in whole animals, and while evidence for tissue-specific differences in control of *Wolbachia* density have been reported (McGraw *et al.* 2002; Osborne *et al.* 2012; Martinez *et al.* 2015; Amuzu and McGraw 2016), introgression or transinfection experiments that would differentiate between cytoplasmic *vs.* nuclear genetic influence on such tissue-specific density differences are lacking. It is also possible that the nature of the cytonuclear interactions that impact *Wolbachia* density differ depending on the time the *Wolbachia* and host have been interacting. Over long evolutionary times, like between *w*Pip and *C*. *pipiens* or *C*. *quinquefasciatus*, *Wolbachia* and its host are likely to coevolve toward mutualism (Weeks *et al.* 2007), perhaps resulting in interactions that generally lower symbiont densities; while during much shorter time frames, like between *w*Mel and *Ae*. *aegypti*, interactions may be more characteristic of parasitism with correspondingly different host responses (Kambris *et al.* 2009; Pan *et al.* 2012; Rances *et al.* 2012).

### Mosquito QTL affecting Wolbachia density in nongonadal tissues

We identified two major-effect QTL affecting variation in *Wolbachia* density in nongonadal tissues of *C*. *quinquefasciatus* (Figure 4). The presence of the QTL was predicted by the introgression analysis that indicated that cytonuclear epistasis determine *Wolbachia* density in nongonadal tissues (Figure 2B). The fact that the QTL explain only ∼23% of phenotypic variance suggests that other QTL with minor effects or those demonstrating nonadditive, epistatic interactions likely remain to be identified. Repeating the QTL analysis with greater statistical power (more mapping individuals and greater recombination density) will allow additional minor-effect and epistatic QTL to be identified with statistical confidence.

Genetic-effect analysis suggested that the polymorphisms underlying the major-effect QTL likely include recessive alleles that are homozygous in the Ben95 strain of *C*. *quinquefasciatus* (Figure 4B). Recessive alleles are most often produced by loss-of-function mutations, which if true in this case, would suggest that the genes that are mutated in Ben95 mosquitoes normally suppress levels of *Wolbachia* in Arg12 mosquitoes. Identifying the genes affected by the causal QTL polymorphisms, and ultimately knowing the molecular pathways involved, would provide insight into *Wolbachia*–host interactions that determine *Wolbachia* density in host tissues. A wide variety of possible molecular pathways for such interactions can be envisioned, from host innate immunity or metabolic pathways that directly impact bacterial density to different aspects of host cell biology that might indirectly modulate *Wolbachia* density, such as rates of autophagy, proteolysis, or pathways involved in movement of *Wolbachia* between tissues (Frydman *et al.* 2006; Voronin *et al.* 2012; White *et al.* 2017a,b).

Identifying the specific polymorphisms underlying the QTL will require both higher resolution genetic linkage maps and improved physical maps of the *C*. *quinquefasciatus* chromosomes. Like many genomes sequenced in recent years purely by a shotgun-sequencing approach, the current *C*. *quinquefasciatus* reference genome is highly fragmented, consisting of 3171 scaffolds (Arensburger *et al.* 2010; see more recently Dudchenko *et al.* 2017). High resolution genetic maps, such as those presented here, are useful for improving genome assemblies (Fierst 2015). We were able to position 435 scaffolds on the physical map, accounting for 14% of the scaffolds by number and 44% of the genome by sequence content (Figure S2, File S2, and Table S2). Even higher resolution genetic linkage maps will be needed, however, to improve the *C*. *quinquefasciatus* genome assembly to a level of accuracy allowing for routine extrapolation between the genetic and physical maps for each chromosome.

The increase in genetic marker resolution reported here also resulted in identification of many inconsistencies between the genome scaffold assemblies in the reference genome and our genetic linkage maps. Discontinuities were identified in 38% of the scaffolds that contained two or more markers, with discontinuous scaffolds being split both within and between LGs (Figure S3, File S2, and Table S2). These inconsistencies could reflect either errors during assembly of the scaffolds in the reference genome, or true chromosomal rearrangements between the Johannesburg strain of *C*. *quinquefasciatus* used to generate the reference genome (Arensburger *et al.* 2010) and the Arg12 and Ben95 strains used here to create the genetic mapping population. Ultimately, an accurate, contiguous physical map of the *C*. *quinquefasciatus* genome, minimally across the DNA sequences genetically delineated by each QTL, will be needed before an accurate collection of candidate polymorphisms can be identified for each QTL.

## Supplementary Material

Supplemental material is available online at www.g3journal.org/lookup/suppl/doi:10.1534/g3.117.043422/-/DC1.
