# Supplementary material for: Cytonuclear Epistasis Controls the Density of Symbiont Wolbachia pipientis in Nongonadal Tissues of Mosquito Culex quinquefasciatus
Source: G3 (Bethesda). 2017 Jun 9;7(8):2627–35. doi: 10.1534/g3.117.043422 (PMC5555468; doi:10.1534/g3.117.043422)
Supplement: Supplementary file 1 [file 2627FileS1.docx]

**SI Materials and Methods**

**Genetic mapping panel.** Because the Arg12 strain of *Cx*. *quinquefasciatus* had only recently been established from a pool of field-collected mosquitoes at the time of these experiments, an isogenized sub-strain generated from a single female that was selected for producing consistently low densities of *Wolbachia* in non-gonadal tissues among her progeny was created and used for linkage analysis. The Ben95 *Cx*. *quinquefasciatus* mosquitoes were not isogenized because they have been subject to decades of laboratory inbreeding. To create F2 hybrids between the isogenized Arg12 and Ben95 strains of *Cx*. *quinquefasciatus* and containing Arg12-derived cytoplasm, 10 Arg12 virgin females were crossed to a single Ben95 male. After mating, females were fed on chicken blood, and the single parental male collected for later genotyping analysis. Blood-fed females were held for one week, and then placed individually into oviposition tubes. A single fertile female was chosen as the parental female of the linkage mapping cross and was collected for later genotyping analysis. The F1 larvae produced by the parental female were reared to adulthood, and the F1 sibling adults allowed to mate *inter* *se* before being fed on chicken blood. F2 egg rafts were collected, and the larvae reared to adulthood. Females used for genetic mapping were randomly chosen from the F2 population when they were 3-5 days old. Each F2 female was ovariectomized, and the carcass collected and stored for later genotyping and phenotype analysis. DNA was isolated from the parental male and female and each ovariectomized F2 hybrid and used for nextRAD sequencing and SNP genotyping. DNA from each ovariectomized F2 hybrid was also used to measure *Wolbachia* density in non-gonadal tissues as described previously (Micieli and Glaser 2014).

**Linkage Mapping.** Linkage mapping was performed using Rqtl (Broman *et al.* 2003; Broman and Sen 2009; Broman 2015). Marker order was first determined using a modified *orderMarkers* function that invoked a *ripple* function after the addition of every 10 markers (R Code S1). Some manual curation of the marker order was performed to optimize the likelihood of the resulting linkage groups. This primarily involved 14 markers that were not automatically placed into one of the three linkage groups. These additional markers were, based on recombination fractions, assigned to a linkage group, and then the function *tryallpositions* was used to find the proper position of that marker in the linkage map. If the likelihood of the genotypes on the new linkage group containing the marker was not significantly less than the likelihood of genotypes on the linkage group not containing the marker, the marker position was retained.

**Supplemental Figure legends**

**Figure S1** *Wolbachia* densities in F2 hybrids of the mapping population. *Wolbachia* densities were measured in ovariectomized females (non-gonadal tissues) of a sample of the F2 hybrids used for QTL mapping and compared to densities in the parental Ben95 (Ben) and Arg12 (Arg) strains of *Cx*. *quinquefasciatus*. The parental lines (P) and F2 hybrids (F2) are indicated across the top of the graph, and the cytoplasmic and nuclear genotypes for each group of ovariectomized mosquitoes is indicated across the bottom of the graph. Medians are indicated by solid horizontal lines, and the assay limit of detection is indicated by a dashed horizontal line.

**Figure S2** Scaffold placement on the *Cx*. *quinquefasciatus* genetic linkage maps. The scaffolds identified at each marker position on the genetic linkage maps are shown as boxes either above the SNP-based linkage maps reported here or below the microsatellite-based linkage maps reported previously (indicated as Hickner et al) (Hickner *et al.* 2013). Discontinuous scaffolds that map to multiple locations on the same (red) or different (blue) linkage groups are indicated. Solid lines between the maps connect markers located on the same genomic scaffolds shared between the two maps. Dashed lines between the maps indicate multiple potential connections due to discontinuous scaffolds that map to multiple locations on the linkage group (see Fig. S3). Connections that optimized agreement in the linear order of markers between the maps were chosen for the maps illustrated in Fig. 3. The genetic maps for the three linkage groups are shown at the same scale with their cM sizes indicated.

**Figure S3** Locations of discontinuous scaffolds. Only scaffolds containing two or more SNP markers and therefore potentially able to be identified as discontinuous are shown relative to the genetic linkage maps. Each upward bracket below the linkage group maps indicates the multiple positions at which a given scaffold was mapped within the same linkage group (red-boxed scaffolds). Each vertical tic along a bracket indicates a different location where the same scaffold was positioned. The lines connecting different linkage groups indicate the multiple positions where a given scaffold was mapped across linkage groups (blue-boxed scaffolds).

**Supplemental Literature Cited**

Broman, K., 2015 R/qtlcharts: interactive graphics for quantitative trait locus mapping. Genetics 199: 359-361.

Broman, K., and S. Sen, 2009 A guide to QTL mapping with R/qtl. Springer, New York.

Broman, K., H. Wu, S. Sen and G. Churchill, 2003 R/qtl: QTL mapping in experimental crosses. Bioinformatics 19: 889-890.

Hickner, P. V., A. Mori, D. D. Chadee and D. W. Severson, 2013 Composite linkage map and enhanced genome map for *Culex* *pipiens* complex mosquitoes. J. Hered. 104: 649-655.

Micieli, M. V., and R. L. Glaser, 2014 Somatic *Wolbachia* (Rickettsiales: Rickettsiaceae) levels in *Culex* *quinquefasciatus* and *Culex* *pipiens* (Diptera: Culicidae) and resistance to West Nile virus infection. J. Med. Entomol. 51: 189-199.
