# Supplementary figures and images for: Cytonuclear Epistasis Controls the Density of Symbiont Wolbachia pipientis in Nongonadal Tissues of Mosquito Culex quinquefasciatus
Source: G3 (Bethesda). 2017 Jun 9;7(8):2627–35. doi: 10.1534/g3.117.043422 (PMC5555468; doi:10.1534/g3.117.043422)

## Slide 1
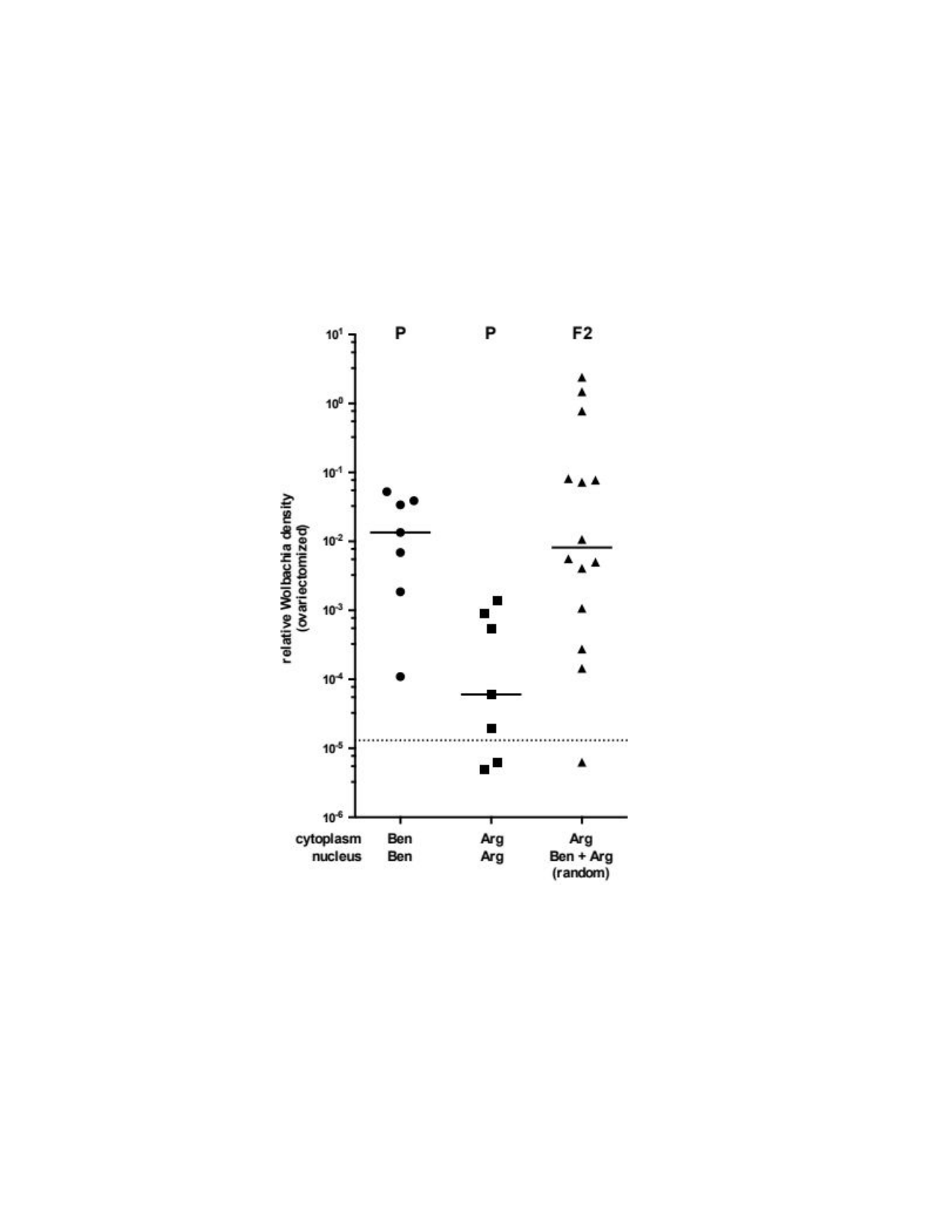

Supplement: Supplementary file 5 [file 2627FigureS1.pptx]

## Slide 1
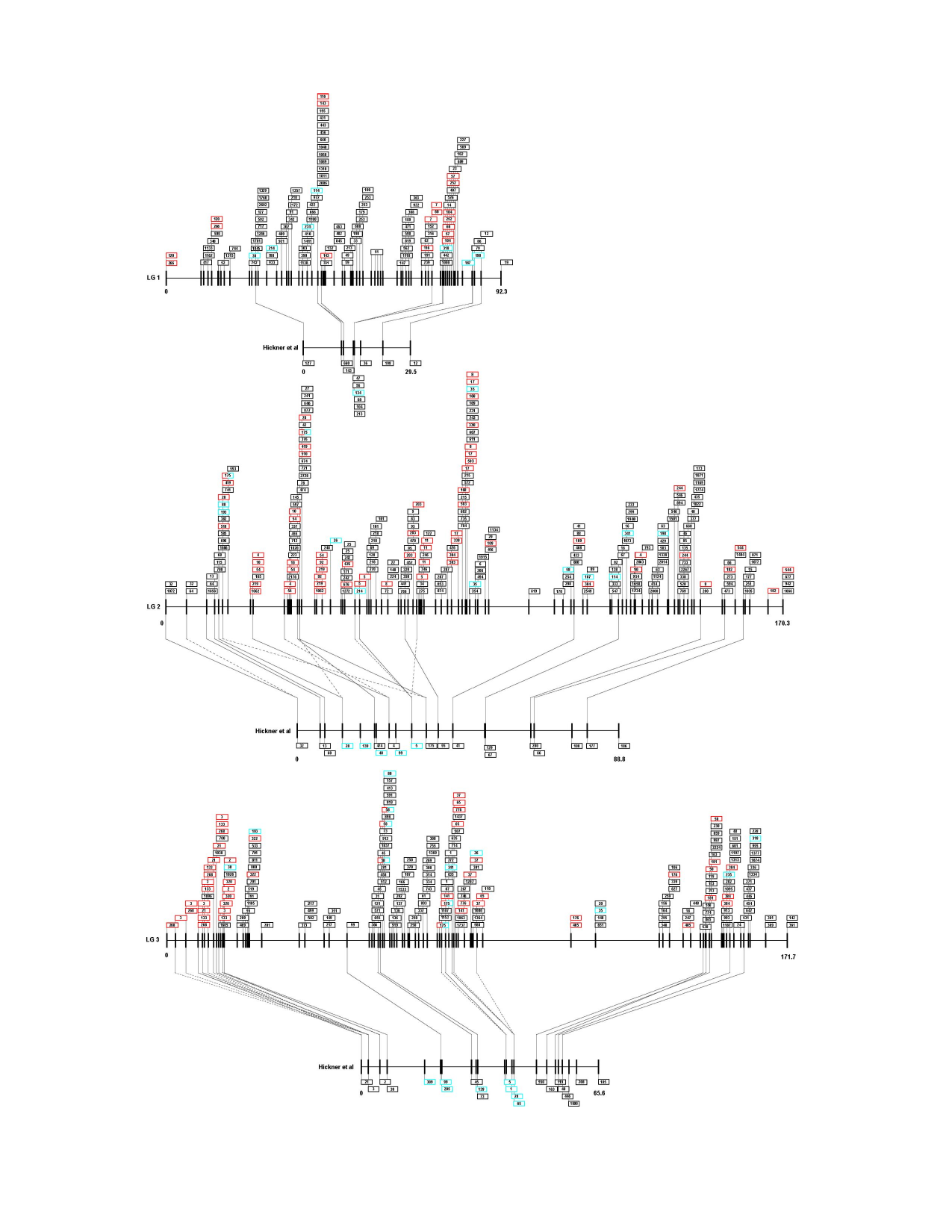

Supplement: Supplementary file 6 [file 2627FigureS2.pptx]

## Slide 1
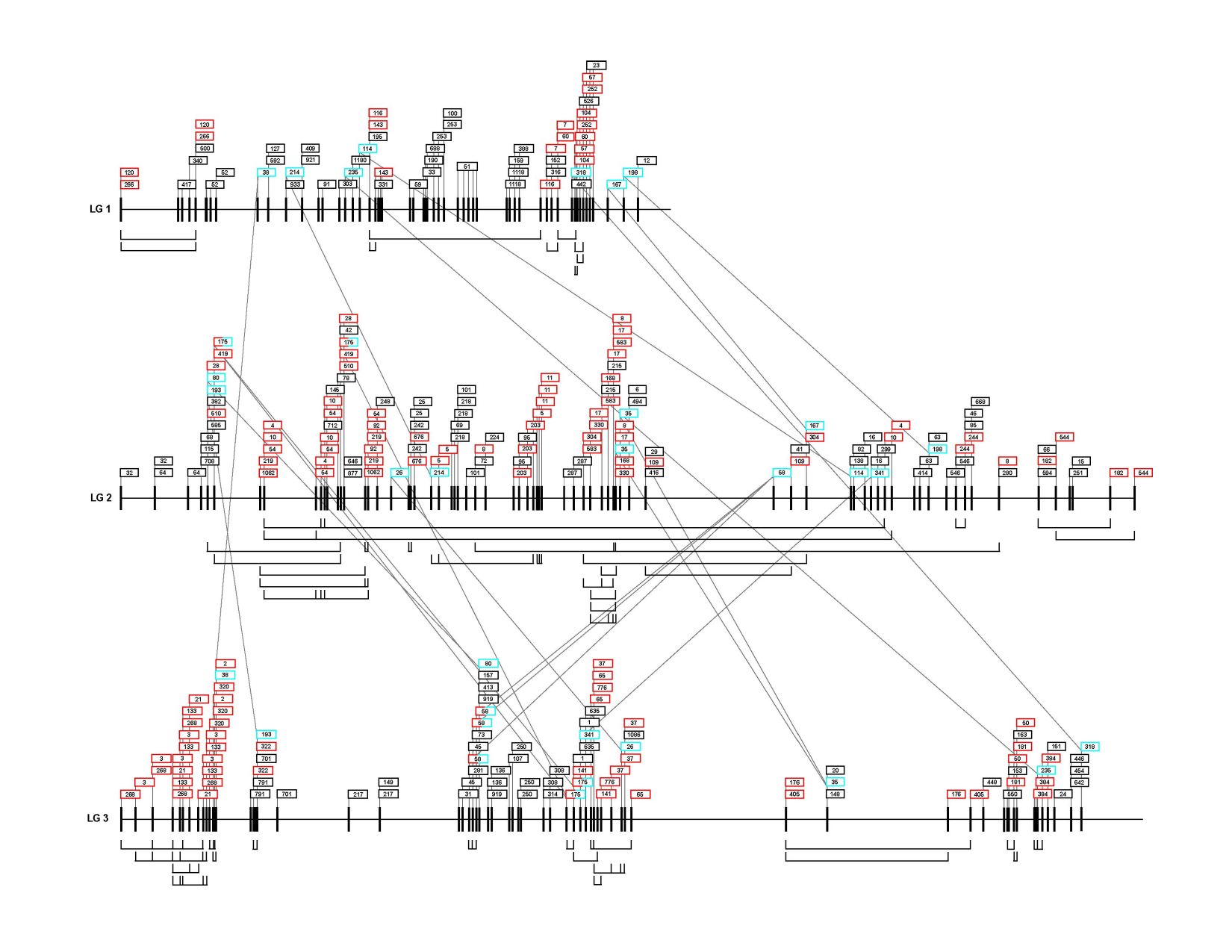

Supplement: Supplementary file 7 [file 2627FigureS3.pptx]
