# Supplementary material for: Cytonuclear Epistasis Controls the Density of Symbiont Wolbachia pipientis in Nongonadal Tissues of Mosquito Culex quinquefasciatus
Source: G3 (Bethesda). 2017 Jun 9;7(8):2627–35. doi: 10.1534/g3.117.043422 (PMC5555468; doi:10.1534/g3.117.043422)
Supplement: Supplementary file 8 [file 2627TableS1.docx]

| **Table S1. ANOVA analysis of introgression results** | | |  |  |  |
| --- | --- | --- | --- | --- | --- |
|  |  |  |  |  |  |
| Source of Variation | df | SS | MS | F | P |
| Gonadal *Wolbachia* Density |  |  |  |  |  |
| nuclear genotype | 1 | 0.597 | 0.597 | 3.388 | 0.07810 |
| cytoplasmic genotype | 1 | 3.976 | 3.976 | 22.569 | 0.00008 |
| cytonuclear interaction | 1 | 0.268 | 0.268 | 1.522 | 0.22920 |
| Residuals | 24 | 4.229 | 0.176 |  |  |
|  |  |  |  |  |  |
| Non-Gonadal *Wolbachia* Density | |  |  |  |  |
| nuclear | 1 | 9.542 | 9.542 | 11.833 | 0.00214 |
| cyto | 1 | 15.668 | 15.668 | 19.428 | 0.00019 |
| nuclear:cyto | 1 | 3.893 | 3.893 | 4.827 | 0.03791 |
| Residuals | 24 | 19.355 | 0.806 |  |  |
