# Supplementary material for: Cytonuclear Epistasis Controls the Density of Symbiont Wolbachia pipientis in Nongonadal Tissues of Mosquito Culex quinquefasciatus
Source: G3 (Bethesda). 2017 Jun 9;7(8):2627–35. doi: 10.1534/g3.117.043422 (PMC5555468; doi:10.1534/g3.117.043422)
Supplement: Supplementary file 9 [file 2627TableS2.docx]

| **Table S2. Linkage Group Statistics** |  |  |  |  |  |
| --- | --- | --- | --- | --- | --- |
|  | Linkage Group | | |  |  |
|  | 1 | 2 | 3 | Total |  |
| Number of nextRAD markers | 203 | 309 | 267 | 779 |  |
| Number of bin markers | 68 | 93 | 85 | 246 |  |
| length (cM) | 92.3 | 170.3 | 171.7 | 434.3 |  |
| Dispersion (P value) | 1.45 (0.68) | 0.77 (0.82) | 0.77 (0.81) |  |  |
|  |  |  |  |  |  |
| Number of scaffolds on map | 108 | 187 | 154 | 435^a^ |  |
| Percent of scaffolds in study | 25% | 43% | 35% | 100% |  |
| Percent of total scaffolds in reference | 3% | 6% | 5% | 14% |  |
| Number of bp in scaffolds | 60,126,812 | 118,109,503 | 89,411,246 | 254,724,796^a^ |  |
| Percent of bp in study | 24% | 46% | 35% | 100% |  |
| Percent of total bp in reference | 10% | 20% | 15% | 44% |  |
| Discontinuous scaffolds^b^ | 16/45 (36%) | 34/77 (44%) | 26/57 (46%) | 62/165 (38%) |  |
| ^a^ Non-redundant total |  |  |  |  |  |
| ^b^ Number of discontinuous scaffolds/number of scaffolds in study with two or more nextRAD markers (percent discontinuous) | | | | |  |
