# Supplementary material for: Cytonuclear Epistasis Controls the Density of Symbiont Wolbachia pipientis in Nongonadal Tissues of Mosquito Culex quinquefasciatus
Source: G3 (Bethesda). 2017 Jun 9;7(8):2627–35. doi: 10.1534/g3.117.043422 (PMC5555468; doi:10.1534/g3.117.043422)
Supplement: Supplementary file 10 [file 2627TableS3.docx]

| **Table S3. QTLs for *Wolbachia* density in non-gonadal tissues** | | | | |
| --- | --- | --- | --- | --- |
|  |  |  |  |  |
| Locus ID | linkage group | position (cM) | scaffold | position (bp) |
| QTL-1 |  |  |  |  |
| 908 | 2 | 0 | 3_1072 | 73998 |
| 10766 | 2 | 0 | 3_32 | 1289154 |
| 10812 | 2 | 0 | 3_32 | 928535 |
| QTL-2 |  |  |  |  |
| 9569 | 2 | 37.6 | 3_27 | 1563604 |
| 8457 | 2 | 37.6 | 3_241 | 273649 |
| 16128 | 2 | 37.6 | 3_646 | 36751 |
| 16130 | 2 | 37.6 | 3_646 | 91790 |
| 18450 | 2 | 37.6 | 3_877 | 123985 |
| 18452 | 2 | 37.6 | 3_877 | 21616 |
| 18453 | 2 | 37.6 | 3_877 | 22712 |
